# An efficient *trans* complementation system for *in vivo* replication of defective poliovirus mutants

**DOI:** 10.1128/jvi.00523-24

**Published:** 2024-06-05

**Authors:** Minetaro Arita

**Affiliations:** 1 Department of Virology II, National Institute of Infectious Diseases, Musashimurayama-shi, Tokyo, Japan; University of Michigan Medical School, Ann Arbor, Michigan, USA

**Keywords:** virus, picornavirus, enterovirus, *trans *complementation

## Abstract

The picornavirus genome encodes a large, single polyprotein that is processed by viral proteases to form an active replication complex. The replication complex is formed with the viral genome, host proteins, and viral proteins that are produced/translated directly from each of the viral genomes (viral proteins provided in *cis*). Efficient complementation *in vivo* of replication complex formation by viral proteins provided in *trans*, thus exogenous or ectopically expressed viral proteins, remains to be demonstrated. Here, we report an efficient *trans* complementation system for the replication of defective poliovirus (PV) mutants by a viral polyprotein precursor in HEK293 cells. Viral 3AB in the polyprotein, but not 2BC, was processed exclusively in *cis*. Replication of a defective PV replicon mutant, with a disrupted cleavage site for viral 3C^pro^ protease between 3C^pro^ and 3D^pol^ (3C/D[A/G] mutant) could be rescued by a viral polyprotein provided in *trans*. Only a defect of 3D^pol^ activity of the replicon could be rescued in *trans*; inactivating mutations in 2C^ATPase/hel^, 3B, and 3C^pro^ of the replicon completely abrogated the *trans*-rescued replication. An intact N-terminus of the 3C^pro^ domain of the 3CD^pro^ provided in *trans* was essential for the *trans*-active function. By using this *trans* complementation system, a high-titer defective PV pseudovirus (PV_pv_) (>10^7^ infectious units per mL) could be produced with the defective mutants, whose replication was completely dependent on *trans* complementation. This work reveals potential roles of exogenous viral proteins in PV replication and offers insights into protein/protein interaction during picornavirus infection.

**IMPORTANCE:**

Viral polyprotein processing is an elaborately controlled step by viral proteases encoded in the polyprotein; fully processed proteins and processing intermediates need to be correctly produced for replication, which can be detrimentally affected even by a small modification of the polyprotein. Purified/isolated viral proteins can retain their enzymatic activities required for viral replication, such as protease, helicase, polymerase, etc. However, when these proteins of picornavirus are exogenously provided (provided in *trans*) to the viral replication complex with a defective viral genome, replication is generally not rescued/complemented, suggesting the importance of viral proteins endogenously provided (provided in *cis*) to the replication complex. In this study, I discovered that only the viral polymerase activity of poliovirus (PV) (the typical member of picornavirus family) could be efficiently rescued by exogenously expressed viral proteins. The current study reveals potential roles for exogenous viral proteins in viral replication and offers insights into interactions during picornavirus infection.

## INTRODUCTION

Picornaviruses are small, non-enveloped viruses with positive-sense, single-stranded RNA genomes (about 6,500 nt [rhinoviruses] to about 9,000 nt [erboviruses]), including poliovirus (PV) as the typical member of this family (*Enterovirus C* species, the genus *Enterovirus,* in the family *Picornaviridae*) (1). The genome of PV encodes a single, large polyprotein (about 2,200 amino acid [aa] residues), which is subsequently processed into each viral protein, and a small protein derived from an upstream open reading frame (uORF) (2). The polyprotein is initially processed into three precursor proteins, P1 (coding VP4VP2VP3VP1), P2 (coding 2A2B2C), and P3 (coding 3A3B3C3D), by viral proteases (2A^pro^ and 3C^pro^/3CD^pro^/3ABC^pro^) (3–5). Subsequently, P1 is processed into viral capsid proteins (VP4VP2[VP0], VP3, VP1), P2 is processed into proteins that have roles in viral RNA synthesis and in virion production/release (2A^pro^ protease, 2B viroporin, 2C^ATPase/hel^ ATPase/helicase) (3, 6–10), and P3 is processed into proteins that most directly serve for RNA synthesis (3A [unknown enzymatic function/recruitment of host proteins GBF1/ACBD3/PI4KB], 3B [also known as VPg, the primer for RNA synthesis], 3C^pro^ proteases, 3D^pol^ polymerase) (1, 11–17). The uORF protein might pose a tissue-specific role in virus growth in gut epithelial cells (2), thus is absent from some members of enterovirus (EV) (i.e., *Enterovirus D* and *Rhinovirus*), which prefer infection in the upper respiratory tract rather than in the gut.

Similar to the completely processed viral proteins, processing intermediates derived from P2 or P3 (i.e., 2BC, 3AB, 3CD^pro^, etc.) are also produced from the polyprotein. Other processing intermediates that span these precursors (e.g., 2C3AB, 2ABC3AB) are produced but only in trace amounts (18, 19). Both processing intermediates and fully processed proteins have critical roles in replication, such as remodeling of the endoplasmic reticulum by 2BC and 3A (20), stimulation of 3D^pol^ activity by 3AB (21–23), efficient cleavage of P1 (4), switching of the viral genome from translation to RNA replication (24, 25), and stimulation of uridylylation of 3B by 3CD^pro^ (26). Disruption of each of the processing intermediate was lethal for infectivity (27), indicating the importance of the processing intermediates in replication.

RNA replication of PV predominantly depends on viral proteins provided in *cis* (28–30). Processing is critically controlled by *cis* cleavage (i.e., cleavage of the polyprotein by 2A^pro^/3C^pro^/3CD^pro^/3ABC^pro^, which are coded in the target polyprotein itself, thus authentic self-cleavage) and by *trans* cleavage (i.e., cleavage of polyprotein by the 2A^pro^/3C^pro^/3CD^pro^/3ABC^pro^, which are coded in polyproteins other than the target polyprotein). Disruptions of the polyprotein downstream of the 2B (27) or introduction of mutations in the 2B2CP3 region without affecting the protease activity (31–35) interfered with *cis* cleavage, resulting in aberrant processing and lethality of virus, underscoring the importance of *cis* cleavage in picornavirus replication and of an intact 2B2CP3 precursor. Recently, involvement of a host PI4KB/OSBP family I (OSBP and OSBP2/ORP4) pathway (16, 36) in processing of 3AB was discovered (37–39). The pathway is essential for the development of a viral replication organelle (RO) (40), formation of the replication complex and synthesis of viral plus-strand RNA (41–43), and enhancement of viral growth and infectivity (44). PI4KB and OSBP family I are the target of anti-EV drug candidates (36, 45–50), suggesting that polyprotein processing is a promising target for antiviral development. Enigmatically, processing of 3AB occurs inefficiently especially in the early phase of replication (51); only 4% of 3AB is processed to provide 3A and 3B for RNA synthesis. Interestingly, resistant PV mutants against PI4KB/OSBP inhibitors have mutations in the 3A region, which enhance the processing of 3AB (37, 40), suggesting that cleavage of 3AB or the final products (3A and 3B) could be a target of exogenous intervention to control infection.

Here, I have developed an efficient *trans*-rescue system for *in vivo* replication of defective PV replicons targeting polyprotein processing. I established cell lines that could conditionally express an entire precursor protein and analyzed the *cis* and *trans* roles of viral proteins. I found that cleavage of 3AB occurs exclusively in *cis* in the polyprotein. Among the defective PV replicon mutants examined, only a mutant (3C/D[A/G]) that has a disrupted cleavage site between 3C^pro^ and 3D^pol^ showed an efficient replication rescued in *trans* and produced pseudovirus at a high titer, comparable to that of the wild-type (WT) replicon. I identified an intact 3CD^pro^ as the minimal protein required for the *trans* rescue.

## RESULTS

### Doxycycline (DOX)-inducible expression of PV non-structural proteins

**Fig 1:**
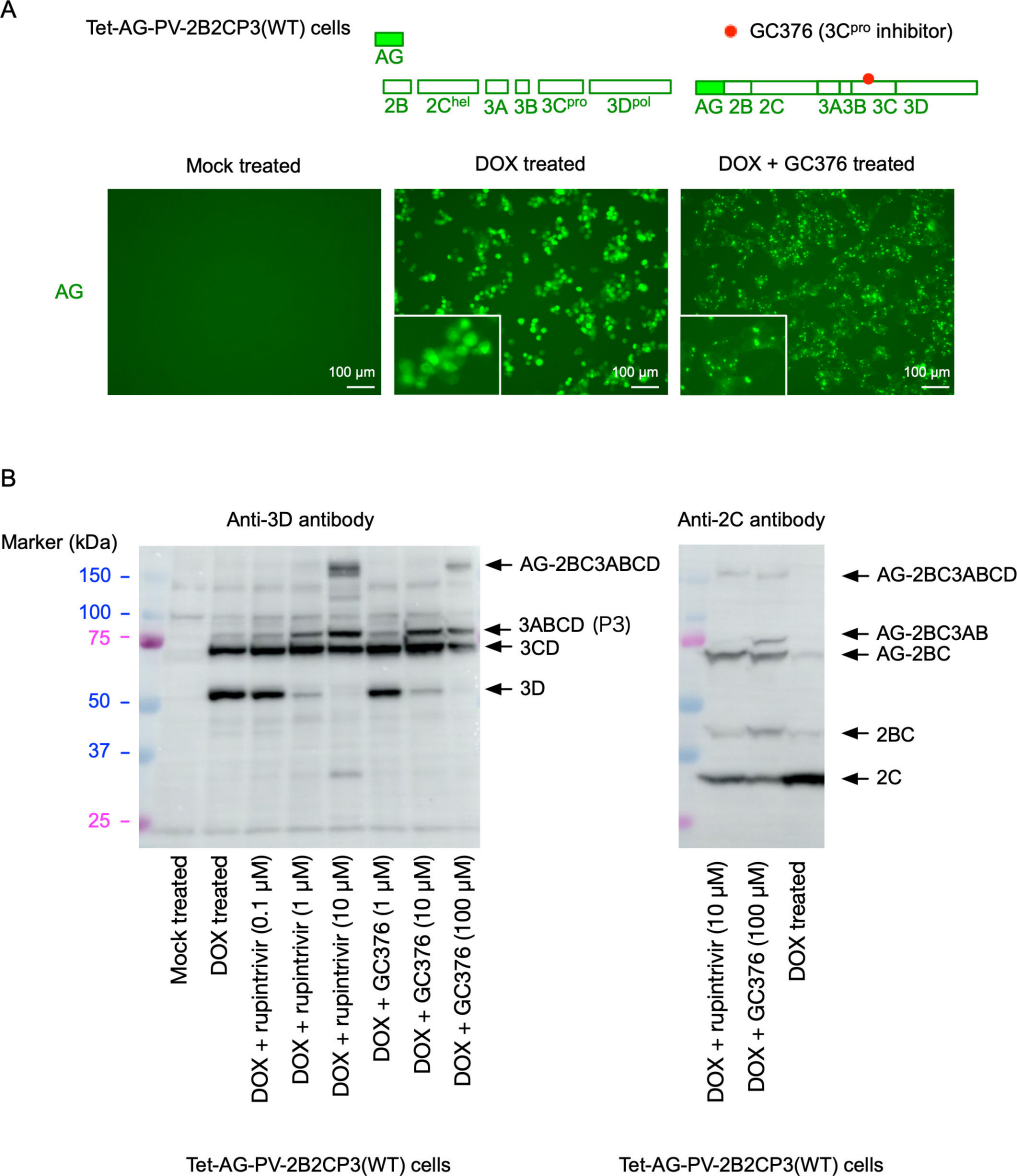
DOX-inducible expression of PV non-structural proteins in Tet-AG-PV-2B2CP3(WT) cells. (**A**) Expression of PV non-structural proteins in Tet-AG-PV-2B2CP3(WT) cells. PV non-structural proteins were expressed as a form of an N-terminally AG-fused single polyprotein in the presence of DOX. The cells were treated with DOX (1 mg/L) for 17 h in the presence or absence of a 3C protease inhibitor GC376 (100 µM). Fluorescent microscope images of the cells with or without DOX treatment are shown. Magnified images of the cells are shown in insets. (**B**) Western blot analysis of the expressed proteins in the cells. Tet-AG-PV-2B2CP3(WT) cells were treated with or without DOX for 17 h in the presence or absence of 3C protease inhibitors GC376 (1, 10, or 100 µM) or rupintrivir (0.1, 1, or 10 µM). Processing intermediates of the polyprotein were detected by anti-3D and anti-2C antibodies.

To analyze the potential role of PV proteins provided in *trans* in replication, I generated a HEK293 cell line (Tet-AG-PV-2B2CP3[WT]) that could express a polyprotein of PV non-structural proteins (2BC3ABCD) in the presence of DOX (1 mg/L) as a form of an N-terminally Azami green (AG)-fused protein, which allowed a high expression level of protein (52) (Fig. 1). Expression of the AG-fused PV polyprotein caused rounded morphology of the cells, similar to the cytopathic effect observed in PV-infected cells. Localization of AG, which is cleaved from the polyprotein by 3C^pro^, was in the nucleus and cytoplasm (Fig. 1A). In the presence of a reversible 3C^pro^ inhibitor GC376 (53), a normal morphology of the cells was retained even after protein expression. In the presence of GC376, AG, which remained attached to the polyprotein, showed a dot-like localization in the cytoplasm, in contrast to that of free AG. Processing of the polyprotein was partially inhibited in the presence of GC376 or rupintrivir (also known as AG7088, an irreversible 3C^pro^ inhibitor) (54), and the polyprotein (161 kDa) remained as an intact precursor (Fig. 1A). In the absence of, or at lower concentrations of, the 3C^pro^ inhibitors, processing intermediates (P3/3ABCD, 3CD^pro^) and a fully processed viral protein (3D^pol^) were detected by an anti-3D^pol^ antibody similar to those present in PV-infected cells (Fig. 1B). These results suggested that Tet-AG-PV-2B2CP3(WT) cells could express a PV polyprotein precursor, which was subsequently processed in the absence of 3C^pro^ inhibitors.

### *trans* rescue of replication of defective PV replicons

**Fig 2:**
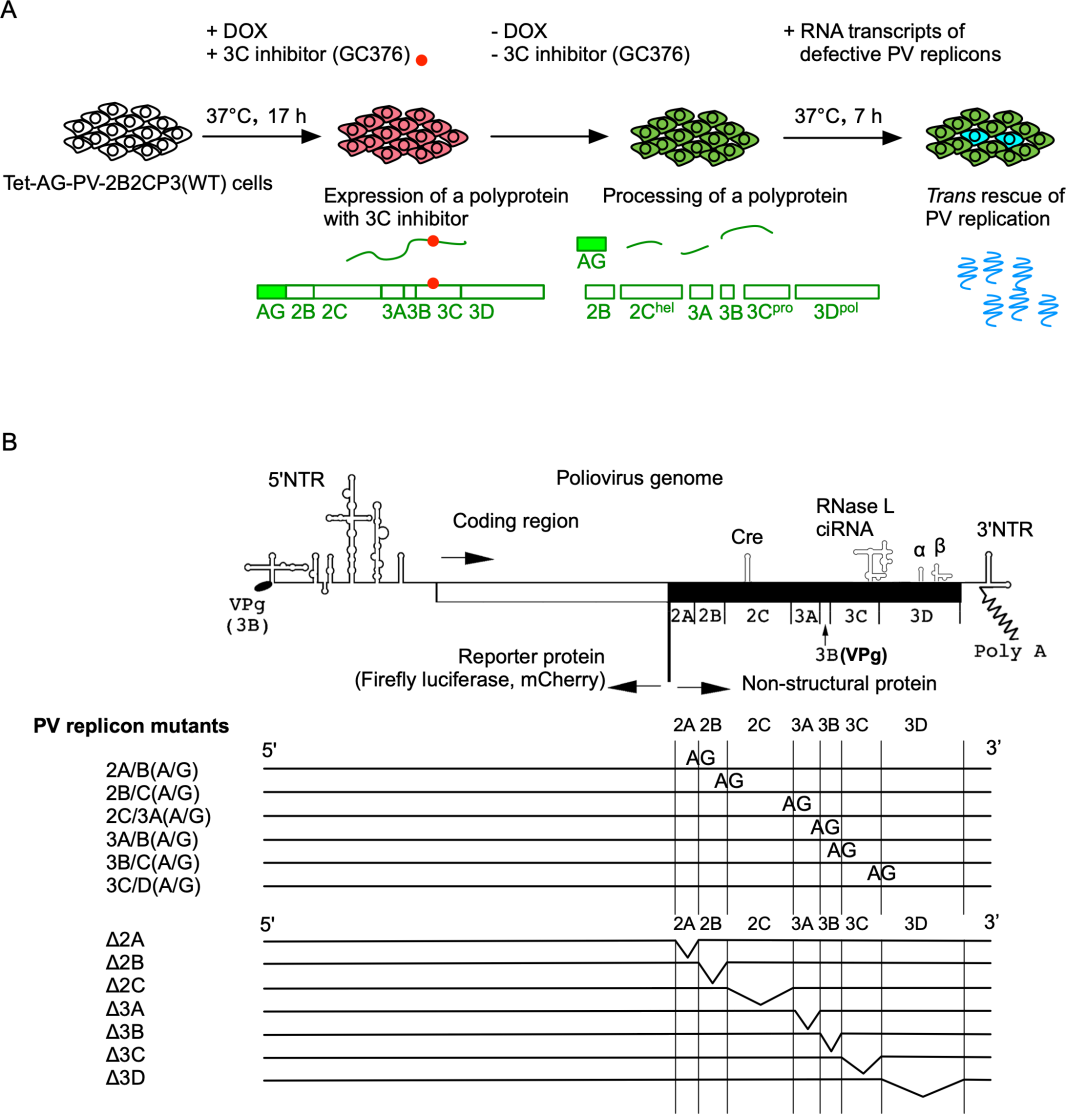
Experimental design of *trans* rescue of replication of defective PV replicons. (**A**) Schematic view of the *trans*-rescue experiment. PV non-structural proteins were expressed in the presence of DOX (1 mg/mL) and GC376 (100 µM) at 37°C for 17 h. Then, RNA transcripts of each PV replicon that has firefly luciferase or mCherry reporter were transfected into cells in the absence of DOX and GC376. The luciferase signals or fluorescence of mCherry in the cells was analyzed at 7 h p.t. of the RNA transcripts. (**B**) Schematic view of PV replicon mutants. Firefly luciferase or mCherry was used as reporters for replication. Introduced amino acid substitutions in the cleavage sites by 3C^pro^ or deletions of each viral gene are shown.

Next, I attempted to *trans* rescue defective PV replicon mutants in Tet-AG-PV-2B2CP3(WT) cells (Fig. 2A). Tet-AG-PV-2B2CP3(WT) cells were first treated with DOX and GC376 to induce expression of the unprocessed polyprotein. The cells were then washed to remove DOX and GC376, and were transfected with RNA transcripts of the defective PV replicon mutants. Replication was monitored by firefly luciferase or mCherry reporters encoded in the replicons. PV replicon mutants that have disrupted cleavage sites (aa substitution of the conserved Q/G to A/G in the cleavage site) for 3C^pro^ between the viral proteins (37, 55, 56), to inhibit the production of fully processed viral proteins (i.e., 2A^pro^, 2B, 2C^ATPase/hel^, 3A, 3B, 3C^pro^, and 3D^pol^), or that lack each of the viral genes were examined (Fig. 2B).

**Fig 3:**
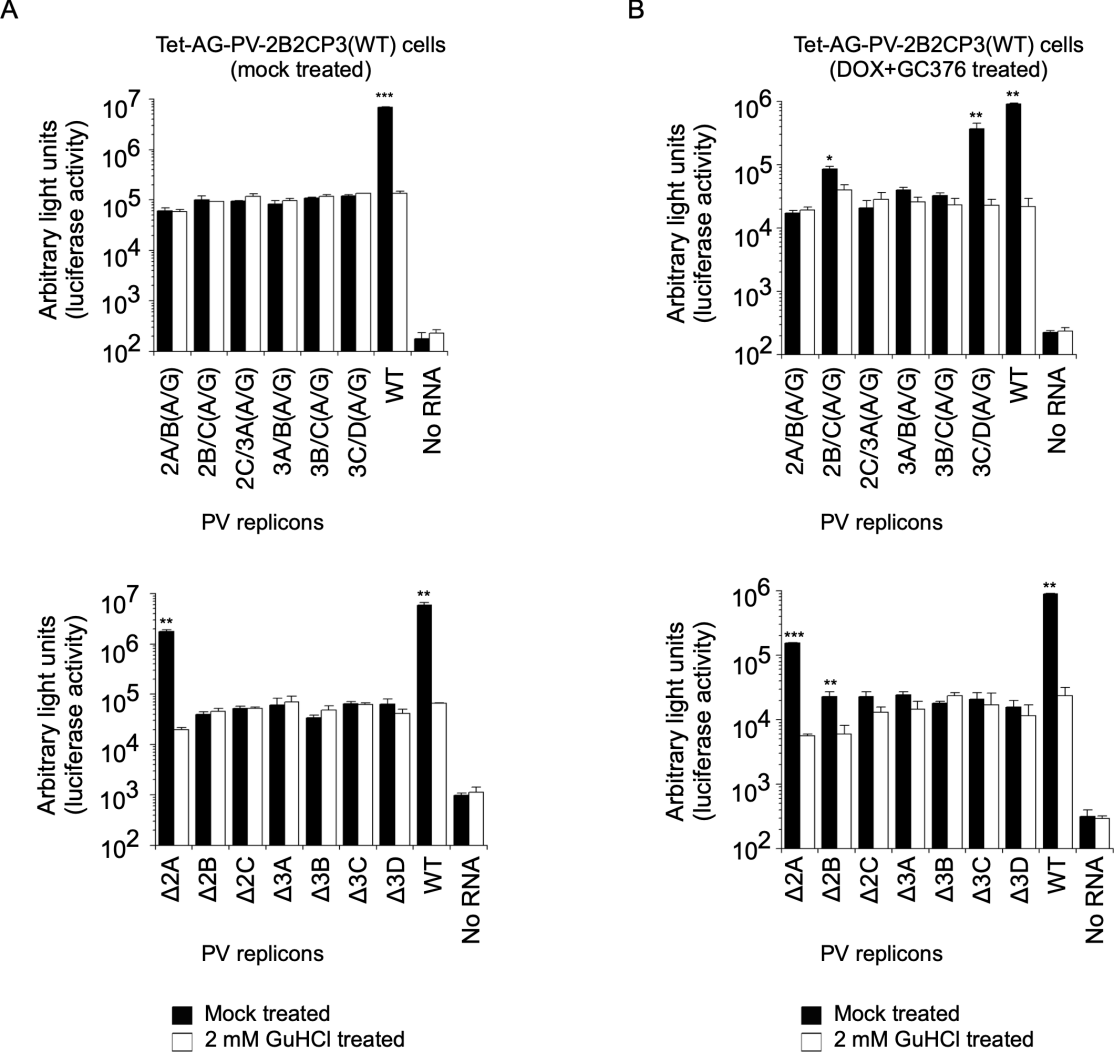
*trans* rescue of replication of defective PV replicons in Tet-AG-PV-2B2CP3(WT) cells. Without DOX treatment (**A**) or after DOX and GC376 treatment (**B**), the cells were transfected with RNA transcripts of each replicon that has firefly luciferase reporter, in the presence or absence of GuHCl (a 2C inhibitor). The luciferase signals measured at 7h p.t. of the RNA transcripts are shown.

In Tet-AG-PV-2B2CP3(WT) cells without DOX treatment, only the WT and Δ2A mutant could replicate (Fig. 3A), consistent with a previous report of a viable 2A^pro^ deletion mutant (57). Other mutants produced only basal levels of signals in the cells, which are derived from initial protein synthesis from transfected RNA transcripts and could not be suppressed in the presence of a PV replication inhibitor guanidine hydrochloride (GuHCl) (a 2C^ATPase/hel^ inhibitor), suggesting no replication occurred. In Tet-AG-PV-2B2CP3(WT) cells treated with DOX and GC376 before RNA transfection, interestingly, a mutant (3C/D[A/G]) could replicate as well as the WT and Δ2A mutant. Replication of 2B/C(A/G) and Δ2B mutants could be detected, albeit at low levels (two- to fourfold increase compared to that in GuHCl-treated cells) (Fig. 3B). This suggested that each viral protein (i.e., 2B, 2C^ATPase/hel^, 3A, 3B, and 3C^pro^) is essential for replication and cannot be *trans* complemented. In addition, replication of a defective PV replicon, which could express 3CD^pro^ but not 3C^pro^ and 3D^pol^, could be efficiently rescued in *trans* by the precursor protein.

### *cis* role of viral proteins in the replication of a PV 3C/D(A/G) mutant

**Fig 4:**
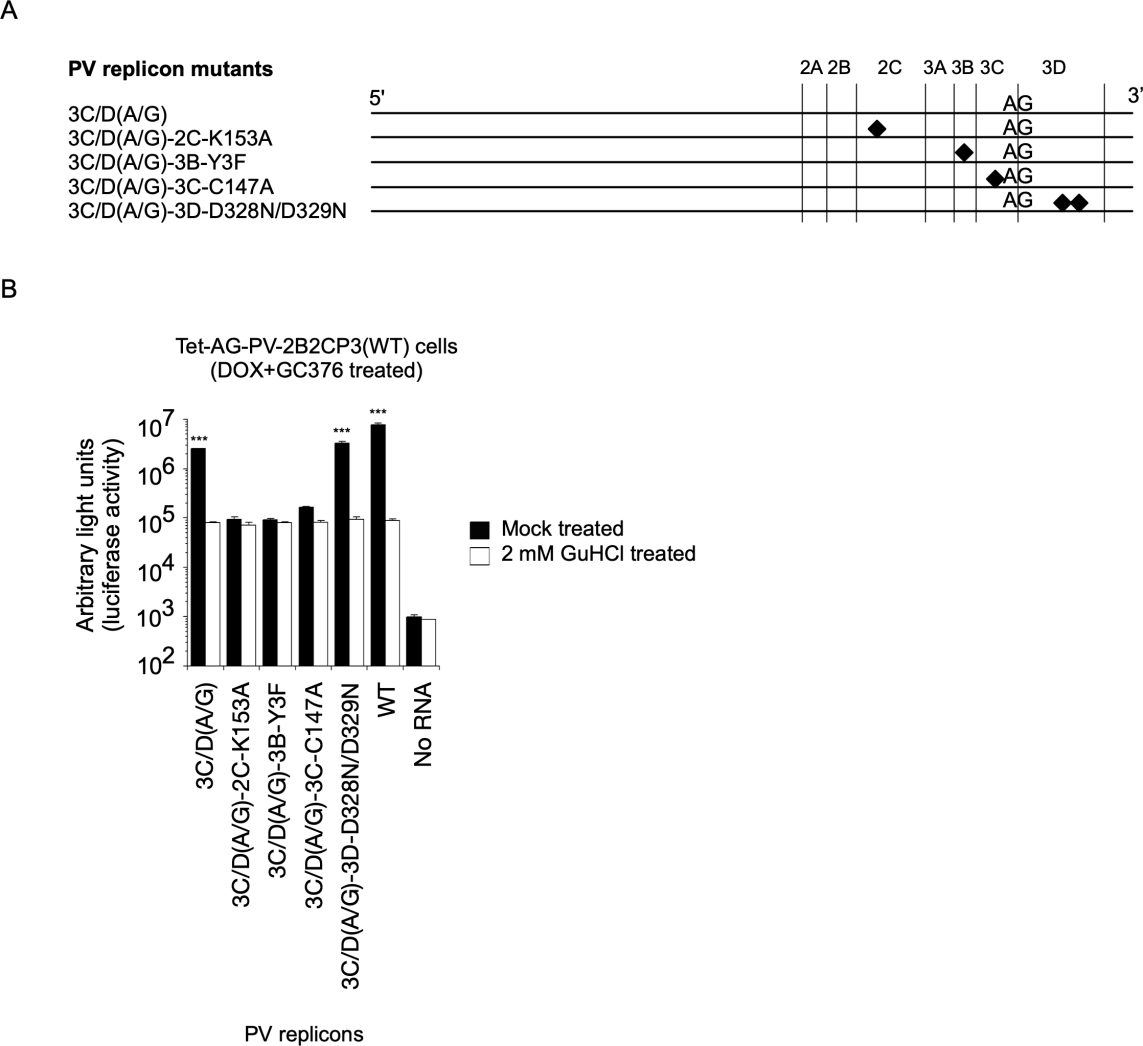
*cis* roles of viral proteins in *trans-*rescued replication of defective PV replicons. (**A**) Schematic view of defective PV replicon mutants with a disrupted cleavage site between the 3C and 3D regions. Firefly luciferase was used as reporter of the replication. (**B**) *trans* rescue of replication of defective PV replicon mutants in Tet-AG-PV-2B2CP3(WT) cells. After DOX and GC376 treatment for 17 h, the cells were transfected with RNA transcripts of each replicon that has firefly luciferase reporter, in the presence or absence of GuHCl. The luciferase signals measured at 7 h p.t. of the RNA transcripts are shown.

Next, to determine the roles of viral proteins provided in *cis* in PV replication, mutations that inactivate activities of viral proteins were introduced into the 3C/D(A/G) mutant (Fig. 4A): 2C-K153A aa substitution that disrupts ATPase activity of 2C^ATPase/hel^ (6), 3B-Y3F aa substitution that inhibits uridylylation of the 3B protein (58), 3C-C147A aa substitution that inactivates the protease activity of 3C^pro^/3CD^pro^ by disruption of the catalytic triad (59), and 3D-D328N/D329N aa substitutions that inactivate the polymerase activity of 3D^pol^ (60). In Tet-AG-PV-2B2CP3(WT) cells treated with DOX and GC376 before RNA transfection, no replication was observed for mutants with inactivated 2C^ATPase/hel^, 3B, or 3C^pro^/3CD^pro^ (Fig. 4B). In contrast, a mutant with inactivated 3D^pol^ (3C/D[A/G]-3D-D328N/D329N) replicated as well as the WT and parental 3C/D(A/G) mutant. This suggested that the polymerase activity of the 3C/D(A/G) mutant can be rescued in *trans* and that *cis* activities of viral proteins (2C^ATPase/hel^, 3B, and 3C^pro^/3CD^pro^) are essential for replication.

### Identification of minimal viral proteins required for *trans* rescue of the replication of defective PV replicons

**Fig 5:**
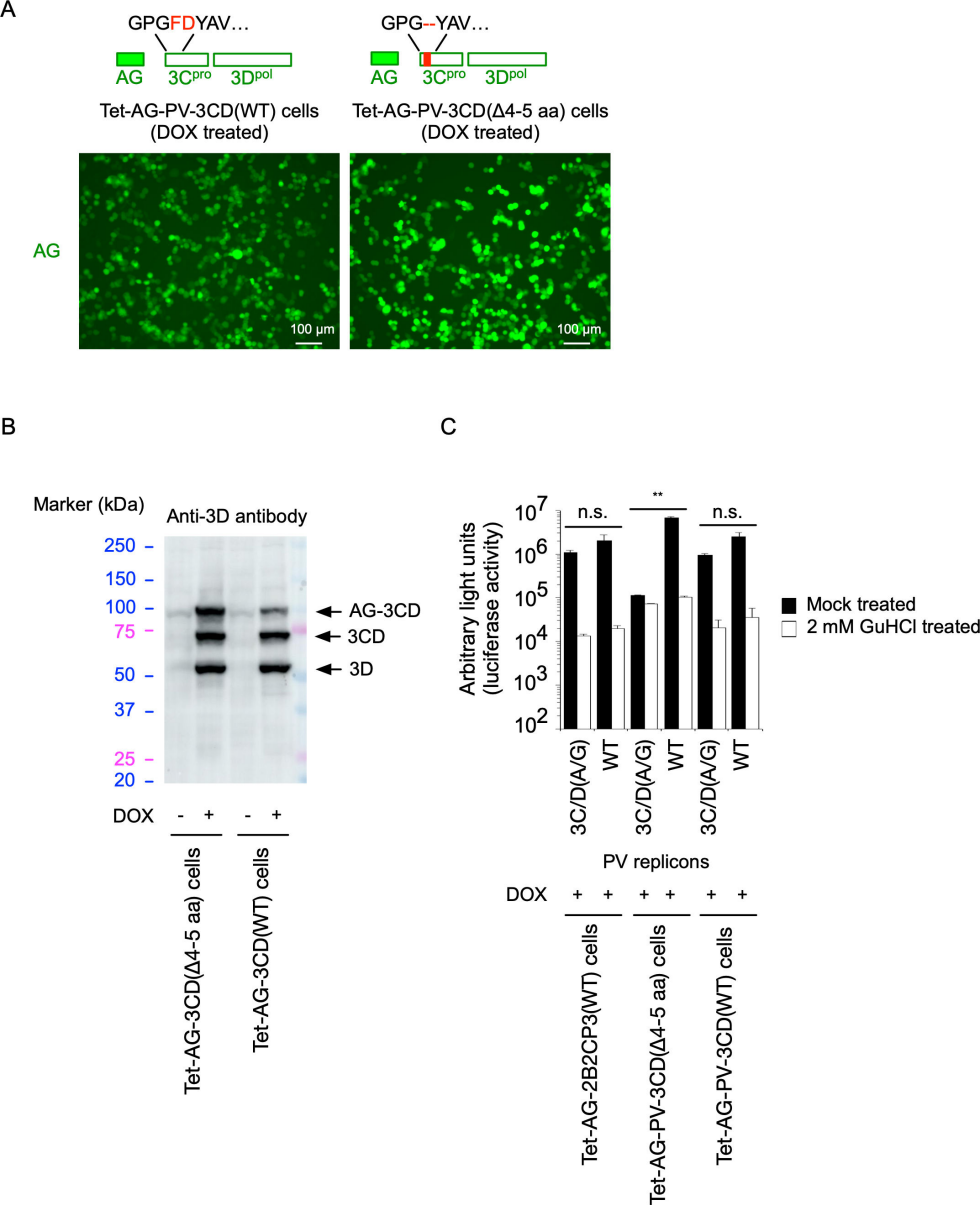
*trans* rescue of replication of defective PV replicons by 3CD^pro^. (**A**) Generation of HEK293 cell lines [Tet-AG-3CD(WT) and Tet-AG-3CD(Δ4–5 aa)] that express PV 3CD proteins (WT or a Δ4–5 aa variant) in the presence of DOX. Fluorescent microscope images of the cells for AG after DOX treatment for 17 h are shown. (**B**) Western blot analysis of the expressed proteins in the cells. The cells were treated with or without DOX for 17 h. Processing intermediates of the 3CD proteins were detected by an anti-3D antibody. (**C**) *trans* rescue of replication of PV 3C/D(A/G) mutant in Tet-AG-3CD(WT) and Tet-AG-3CD(Δ4–5 aa) cells. After the DOX and GC376 treatment for 17 h, the cells were transfected with RNA transcripts of each replicon (WT and 3C/D[A/G] mutant) that has firefly luciferase reporter, in the presence or absence of GuHCl. The luciferase signals measured at 7 h p.t. of the RNA transcripts are shown.

**Fig 6:**
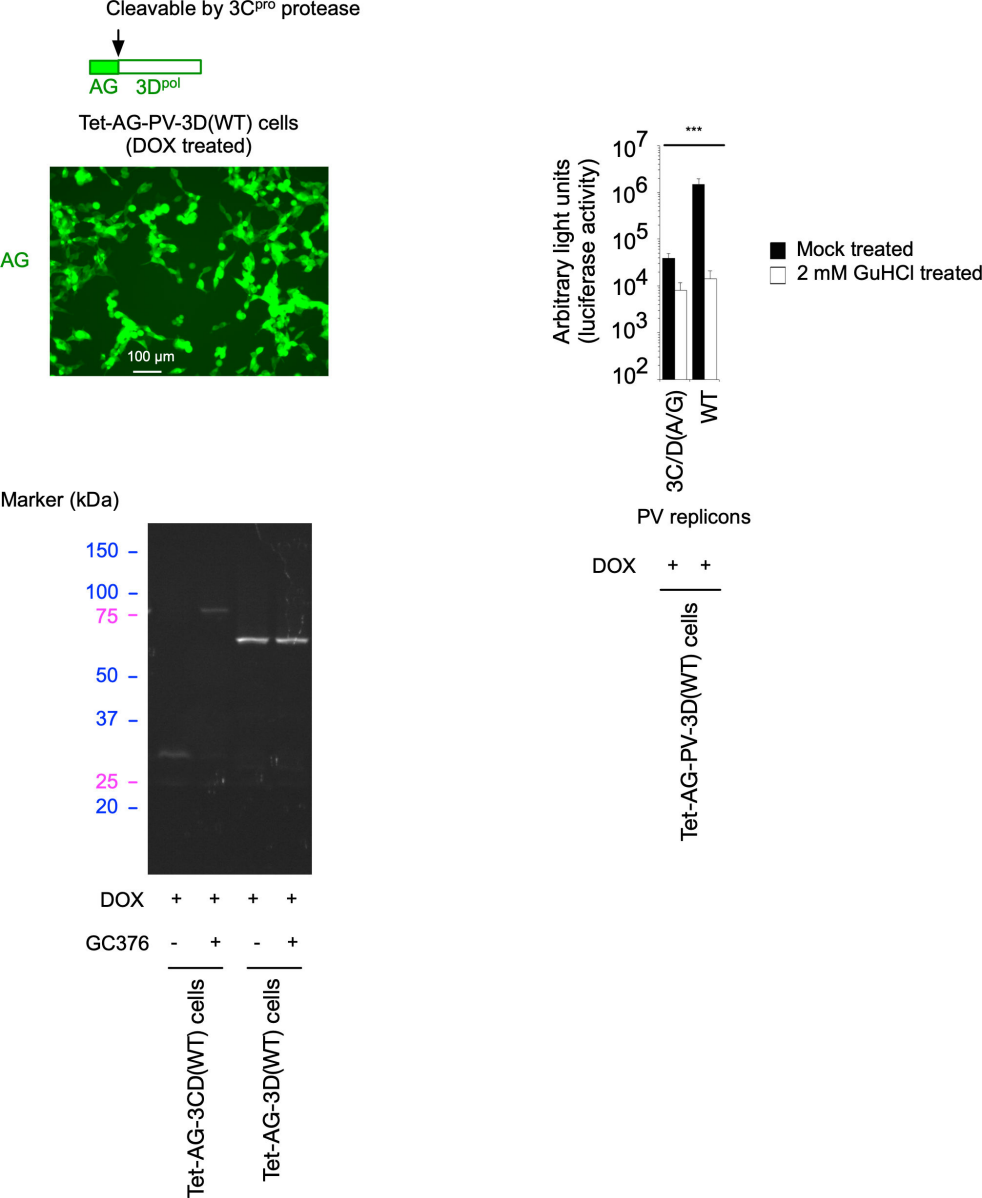
*trans* rescue of replication of a defective PV replicon by PV 3D^pol^ protein. (Top left) Generation of DOX-inducible HEK293 cell lines [Tet-AG-3D(WT)] that express PV 3D^pol^ protein (WT). Fluorescent microscope images of the cells for AG after DOX treatment (for 17 h) are shown. (Bottom left) SDS-PAGE analysis of the expressed proteins in the cells. The cells were treated with or without DOX for 17 h. Fluorescence of AG on the gel is shown. (Right) *trans* rescue of replication of a defective PV replicon in Tet-AG-3D(WT) cells. After the DOX treatment, the cells were transfected with RNA transcripts of each replicon (WT or 3C/D[A/G] mutant) that has firefly luciferase reporter in the presence or absence of GuHCl. The luciferase signals measured at 7 h pt RNA are shown.

**Fig 7:**
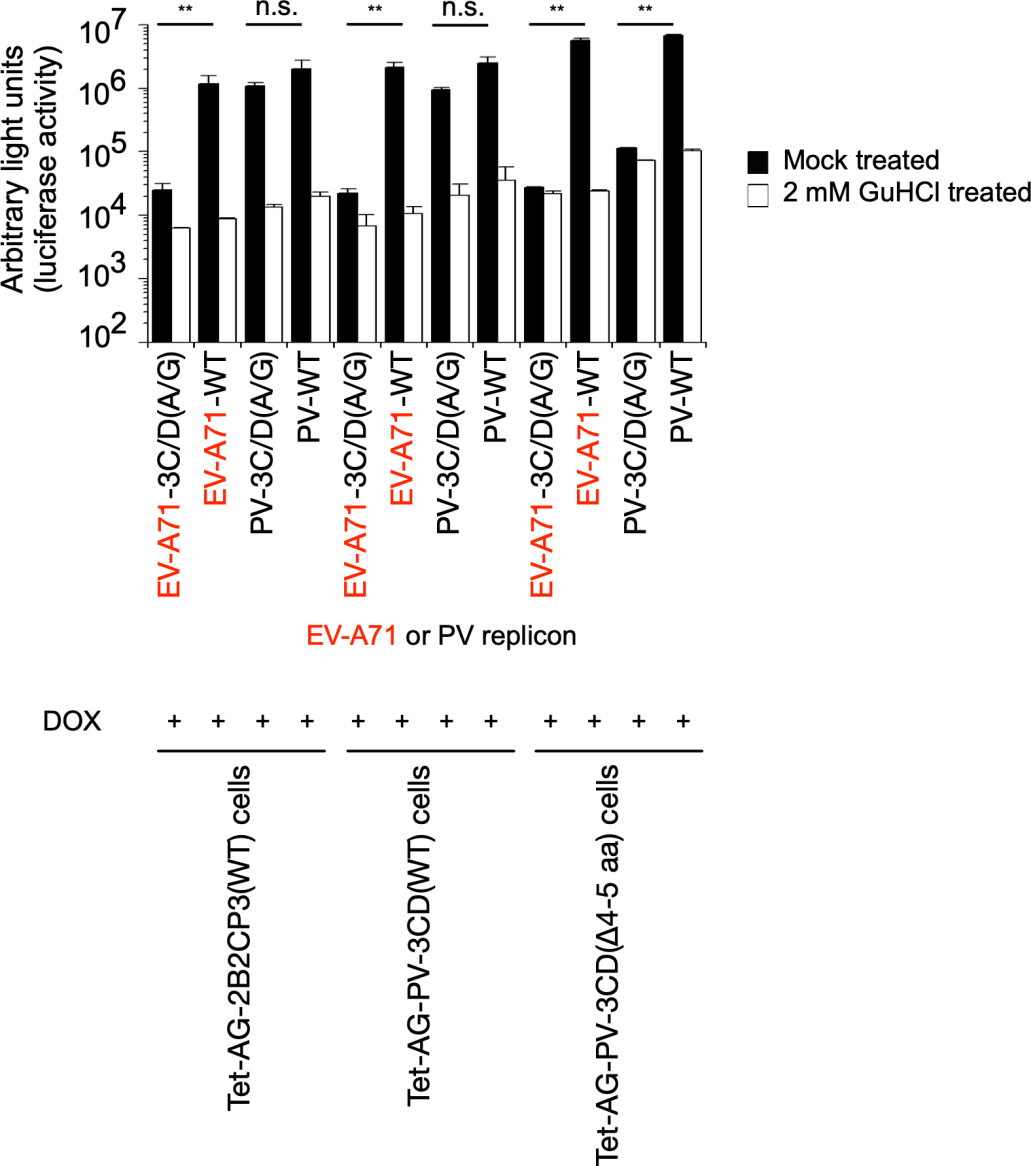
*trans* rescue of replication of a defective EV-A71 or PV replicon. After the DOX and GC376 treatment of indicated cells, the cells were transfected with RNA transcripts of each replicon that has firefly luciferase reporter in the presence or absence of GuHCl (a 2C inhibitor). The luciferase signals measured at 7 h pt RNA are shown.

Because the polymerase activity seemed to be the target of the *trans* rescue of the 3C/D(A/G) mutant, I generated a HEK293 cell line (Tet-AG-PV-3CD[WT]) that expresses PV 3CD^pro^ as a form of an N-terminally AG-fused protein in the presence of DOX, which could be self-processed into 3D^pol^ with an intact N-terminus, which is essential for the polymerase activity (61) (Fig. 5A). Fortuitously, a cell line (Tet-AG-PV-3CD[Δ4-5 aa]) that expresses a 3CD^pro^ variant (deletion of aa 4 and 5 of 3C^pro^, possibly derived from mutations in the oligo DNAs used for the cloning) was also produced. Both cell lines expressed AG-3CD^pro^ and a processing intermediate 3CD^pro^ and 3D^pol^ in the presence of DOX (Fig. 5B). Surprisingly, replication of the 3C/D(A/G) mutant was rescued only in Tet-AG-PV-3CD(WT) cells but not in Tet-AG-PV-3CD(Δ4-5 aa) cells (Fig. 5C), suggesting that an intact 3CD^pro^ is essential and sufficient for the *trans* rescue. Expression of N-terminally AG-fused 3D^pol^, which could be processed by 3CD^pro^ of the 3C/D(A/G) mutant to give an intact 3D^pol^, could only partially rescue the replication of this mutant in *trans* (Fig. 6). The WT replicon replicated to similar levels in both cell lines, suggesting that the effect was specific to the *trans*-rescued replication. To further analyze the specificity of PV 3CD^pro^ provided in *trans,* I attempted to rescue the replication of enterovirus 71 (EV-A71), which belongs to the *Enterovirus A* species, thus another species in EV. A mutation that disrupts the 3C^pro^ cleavage site between 3C^pro^ and 3D^pol^ was introduced in an EV-A71 replicon (EV-A71-3C/D[A/G] mutant), and then replication in cells was analyzed (Fig. 7). In contrast to the PV replicon mutant, replication of the EV-A71-3C/D(A/G) mutant was not rescued in *trans* by a PV polyprotein or 3CD^pro^. These results suggested that the intact 3C^pro^ domain of 3CD^pro^ and viral species-specific interaction of 3CD^pro^ are essential for the *trans*-active function.

### *trans* rescue of replication of defective PV replicons by 3CD^pro^

To clarify the role of 3C^pro^ in the *trans*-active function of 3CD^pro^, I introduced aa substitutions into the 3C^pro^ domain of 3CD^pro^, focusing on those involved in the binding to viral RNA and phospholipids (4, 24, 26, 62). I introduced 3C-R13N, 3C-K82N, and 3C-R84S aa substitutions into 3CD^pro^, which abolish the binding to viral RNA and phospholipids (24, 26, 62–64). I also introduced a mutation to disrupt the 3C^pro^ cleavage site between AG and the 3C^pro^ region to analyze the effect of potential steric hindrance for the interaction with target molecules around the N-terminus of 3CD ^pro^ on the *trans* rescue.

**Fig 8:**
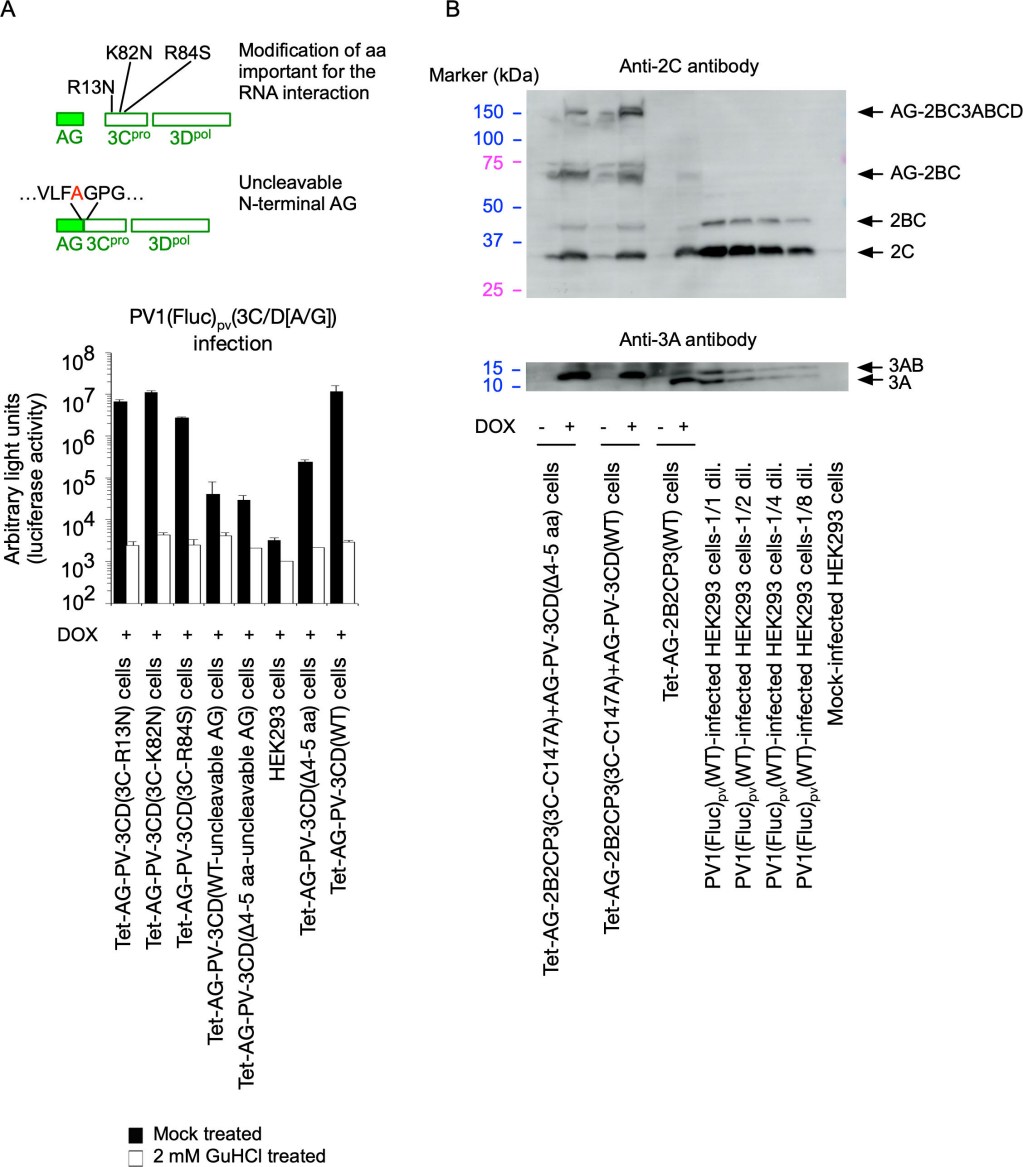
Effect of modifications of 3CD^pro^ on the *trans*-active function. (**A**) Effects of amino acid substitutions that affect the interaction of 3CD with RNA or of N-terminal modification by addition of uncleavable AG on the *trans*-active function. Cells expressing each AG-3CD variant were treated with DOX (1 mg/mL) at 37°C for 5 h and then infected with PV1(Fluc)_pv_(3C/D[A/G]) at a multiplicity of infection (MOI) of 0.05 in the presence or absence of GuHCl. The luciferase signals measured at 17 h post-infection (p.i.) are shown. (**B**) *trans*-cleavage activity of 3CD (WT and a Δ4–5 aa variant). Western blot analysis for the processing intermediates derived from a polyprotein is shown. Cells were treated with or without DOX for 17 h to co-express a polyprotein without the 3C protease activity conferred by a 3C-C147A substitution and AG-3CD (WT or a Δ4–5 aa variant). Processing intermediates derived from the polyprotein were detected by anti-2C or 3A antibodies. Lysates of Tet-AG-2B2CP3(WT) cells and serially diluted lysates of PV1(Fluc)_pv_-infected cells (dilution of 1/1 to 1/16) were taken as positive controls for the processing intermediates.

I generated HEK293 cell lines that could express these 3CD^pro^ variants and analyzed the *trans*-active function for PV1(Fluc)_pv_(3C/D[A/G]) infection (Fig. 8A). Unexpectedly, the 3CD^pro^ variants (3C-R13N, 3C-K82N, and 3C-R84S) efficiently rescued the infection in *trans*, higher than the 3CD(Δ4-5 aa) variant. In contrast, the 3CD^pro^ variants with uncleavable AG could not substantially rescue the infection in *trans* irrespective of the deletion in the N-terminal region of 3C^pro^. This suggested that the integrity of the N-terminus of 3C^pro^, but not the binding activity to viral RNA or phospholipids, is essential for the *trans*-active function of 3CD^pro^.

**Fig 9:**
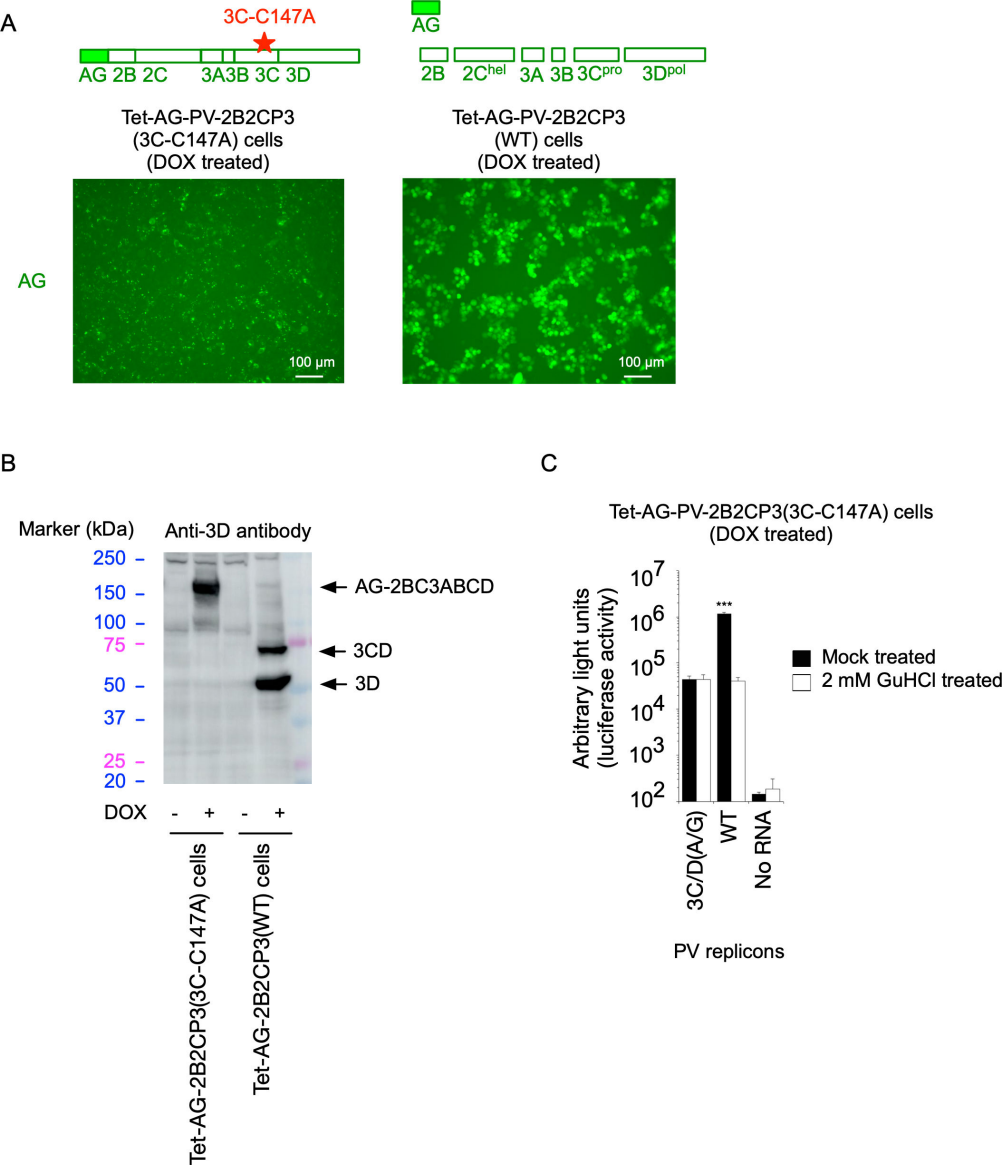
Effect of the 3C^pro^ activity on *trans* rescue of replication of a defective PV replicon. (**A**) Generation of a HEK293 cell line (Tet-AG-PV-2B2CP3[3C-C147A]) that expresses a PV polyprotein without the 3C protease activity conferred by a 3C-C147A substitution in the presence of DOX. Fluorescent microscope images of the cells for AG after DOX treatment (for 17 h) are shown. (**B**) Western blot analysis of the expressed proteins in the cells. The cells were treated with or without DOX for 17 h. Processing intermediates of the polyprotein were detected by an anti-3D antibody. (**C**) *trans* rescue of replication of a defective PV replicon in Tet-AG-PV-2B2CP3(3C-C147A) cells. After DOX treatment for 17 h, the cells were transfected with RNA transcripts of each replicon that has firefly luciferase reporter in the presence or absence of GuHCl. The luciferase signals measured at 7 h p.t. of the RNA transcripts are shown.

To analyze the effect of N-terminal modification of 3CD^pro^ on the *trans* activity, I generated HEK293 cell lines that could simultaneously express 3CD^pro^(WT) or 3CD(Δ4-5 aa) variant with a PV polyprotein (2BC3ABCD), which lacks 3C^pro^ protease activity with a 3C-C147A aa substitution (59) (Tet-AG-PV-2B2CP3[3C-C147A]+AG-PC-3CD[WT] cells and Tet-AG-PV-2B2CP3[3C-C147A]+AG-PC-3CD[Δ4-5 aa] cells) (Fig. 8B). Due to the lack of 3C^pro^ activity, the polyprotein remained intact without producing processing intermediates and could not rescue the replication of the 3C/D(A/G) mutant in *trans* (Fig. 9). Simultaneous expression of this polyprotein with 3CD^pro^(WT) or 3CD^pro^(Δ4-5 aa) variant showed similar profiles of the precursor and processing intermediates AG-2BC, 2BC, 2C, and 3AB; interestingly, no 3A but only 3AB was observed in these cells, suggesting that 3AB is the target of *cis* cleavage (Fig. 8B). Both 3A and 3AB were observed in Tet-AG-PV-2B2CP3(WT) cells or in PV_pv_-infected cells. The 3AB protein was detected in the most diluted lysates of infected cells, at levels lower than those in the Tet-AG-PV-2B2CP3(3C-C147A)+AG-PC-3CD(WT) cells and Tet-AG-PV-2B2CP3(3C-C147A)+AG-PC-3CD(Δ4-5 aa) cells, confirming the absence of 3A in these cells. These results suggested that 3CD^pro^(WT) and 3CD^pro^(Δ4-5 aa) have similar *trans* cleavage activities and that processing of 3AB occurs exclusively in *cis* in the context of the polyprotein.

### Production of PV pseudovirus (PV_pv_) with defective PV replicons

**Fig 10:**
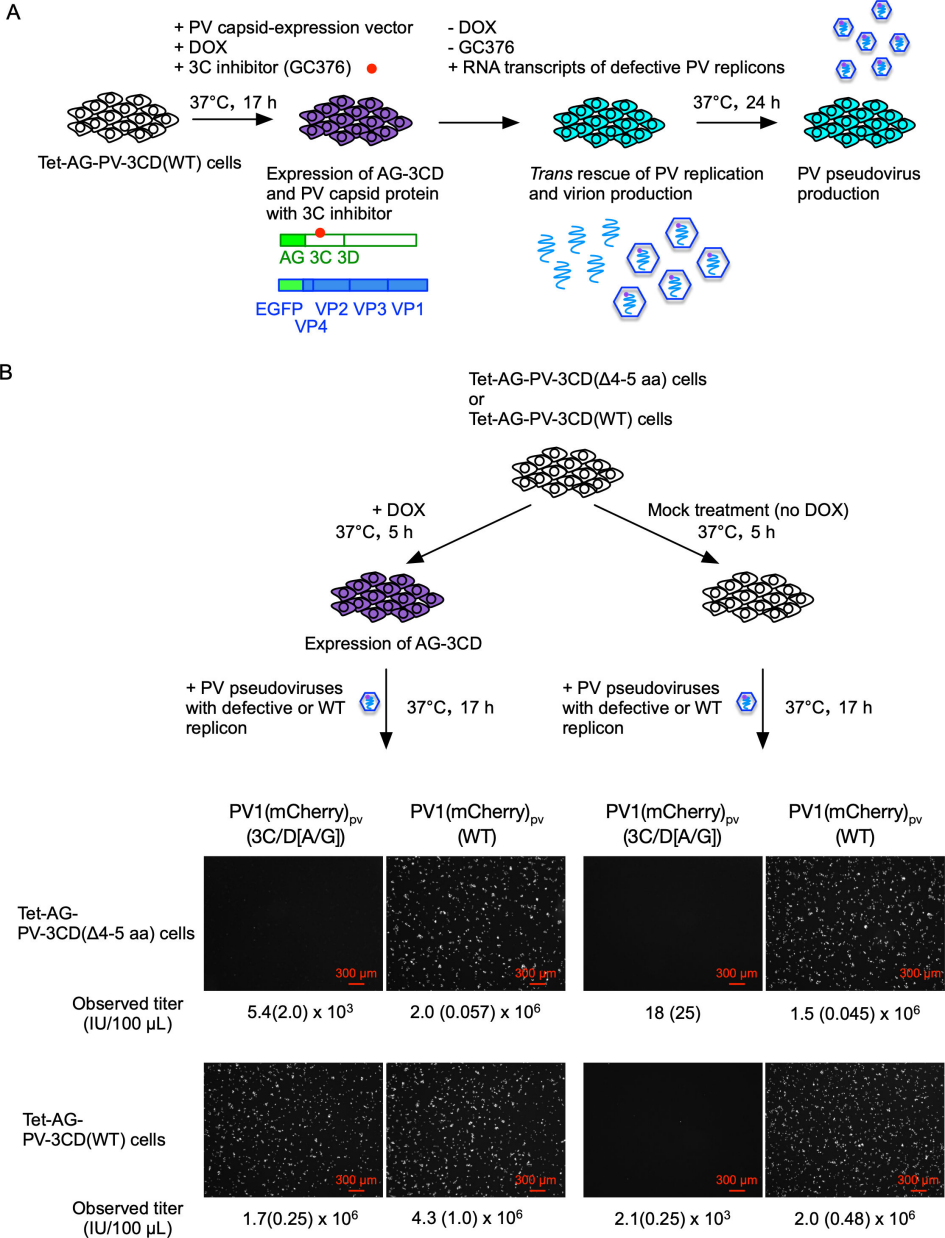
Production of defective PV_pv_. (**A**) Experimental design for *trans* rescue of PV_pv_ production. Tet-AG-3CD(WT) cells were transfected with a PV capsid expression vector in the presence of DOX (1 mg/mL) and GC376 (100 µM) to express the 3CD protein at 37°C for 17 h. Then, RNA transcripts of each PV replicon (WT or 3C/D[A/G] mutant) were transfected to the cells in the absence of DOX and GC376. The cells were collected at around 24 h p.t. of the RNA transcripts. (**B**) Infectivity of PV_pv_ produced with a defective PV replicon with mCherry reporter. Tet-AG-3CD(WT) or Tet-AG-3CD(Δ4–5 aa) cells were treated with or without DOX (1 mg/mL) at 37°C for 5 h. The cells were infected with 10 µL of PV_pv_ solution and then incubated at 37°C for 17h. Fluorescent microscope images of the cells for mCherry and the observed titers of PV_pv_ (IU/100 µL) are shown.

To substantiate the observed high replication level of the defective PV replicons, I attempted to produce PV_pv_ with the defective PV replicons and PV capsid proteins (65) (Fig. 10). Replication-competent PV WT replicon could produce PV_pv_ to a titer (10^7^ to 10^8^ infectious units [IU] per mL) comparable to that of PV virus (65, 66).

**Fig 11:**
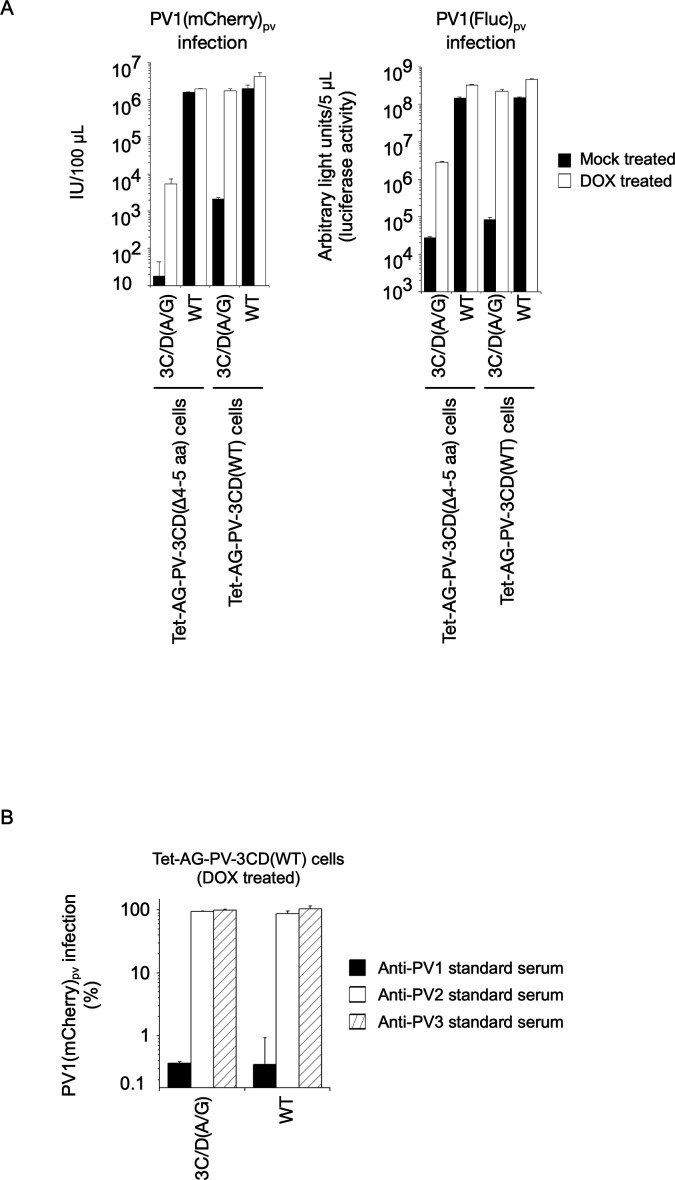
Titer and antigenicity of defective PV_pv_. (**A**) Titer of defective PV_pv_ (3C/D[A/G] mutant) with mCherry or firefly luciferase reporter. Observed titer of PV1(mCherry)_pv_ (IU/100 µL) and luciferase signals in the cells infected with 5 µL of PV1(Fluc)_pv_ are shown. (**B**) Neutralization of PV1(mCherry)_pv_. PV1(mCherry)_pv_ (2.0 × 10^3^ IU) was incubated with standard anti-PV1, PV2, or PV3 antisera (26 U for each type) at 4°C for 7 h, and then added to the DOX-treated Tet-AG-3CD(WT) cells. The number of the cells positive for mCherry fluorescence was counted at 17 h post-infection (p.i.) PV1(mCherry)_pv_ infection in the absence of antisera was taken 100%.

To produce PV_pv_, a plasmid expression vector for type 1 PV capsid proteins was transfected into Tet-AG-PV-3CD(WT) cells in the presence of DOX and GC376 (Fig. 10A). After expression of the PV capsid proteins and AG-PV-3CD(WT), RNA transcripts of the 3C/D(A/G) mutants, with mCherry or firefly luciferase reporters, were transfected into the cells in the absence of DOX and GC376 for the production of PV_pv_ (PV1[mCherry]_pv_[3C/D{A/G}] or PV1[Fluc]_pv_[3C/D{A/G}], respectively). As controls, PV_pv_ was produced with the WT replicons with mCherry or firefly luciferase reporters (PV1[mCherry]_pv_[WT] or PV1[Fluc]_pv_[WT], respectively). Typical cytopathic effects were observed in the cells on day 1 post-transfection (p.t.) of the RNA transcripts, then the cells were harvested to determine the titer of PV_pv_. Collected PV_pv_ was inoculated into Tet-AG-PV-3CD(WT) cells or Tet-AG-PV-3CD(Δ4-5 aa) cells (a control) pre-treated with DOX (or no DOX treatment as a control) before the infection, and then the signals of reporters (fluorescence of mCherry or luciferase activity) in the infected cells were analyzed (Fig. 10B). I observed fluorescence of mCherry in the cells infected with PV1(mCherry)_pv_(WT), irrespective of the DOX treatment and the cell types. In contrast, substantial replication of PV1(mCherry)_pv_(3C/D[A/G]) was observed only in Tet-AG-PV-3CD(WT) cells after pre-treatment with DOX. The observed titer of PV1(mCherry)_pv_(3C/D[A/G]) was about 10^7^IU/mL, thus similar to that of PV1(mCherry)_pv_(WT). Replication in Tet-AG-PV-3CD(Δ4-5 aa) cells was significantly suppressed; about 1/100-fold or 1/100,000-fold lower than that of the WT replicon, in the presence or absence of DOX, respectively (Fig. 11A), supporting the weak *trans*-active function of 3CD^pro^(Δ4-5 aa). Infectivity of PV1(Fluc)_pv_(3C/D[A/G]) showed similar cell-type specificity and dependency on DOX treatment to that of PV1(mCherry)_pv_(3C/D[A/G]). To further confirm the presence of PV_pv_ in the preparations, I performed neutralization tests for PV1(mCherry)_pv_(3C/D[A/G]) with anti-PV antisera (Fig. 11B). PV1(mCherry)_pv_(3C/D[A/G]) was incubated with type-specific anti-PV antibodies (i.e., anti-PV1, PV2, PV3 standard antisera) and then inoculated into Tet-AG-PV-3CD(WT) cells, pre-treated with DOX. Infection of PV1(mCherry)_pv_(3C/D[A/G]) was inhibited by pre-incubation with anti-PV1 antiserum, but not with anti-PV2 or PV3 antisera, suggesting that the type 1 PV antigenicity is retained on PV1(mCherry)_pv_(3C/D[A/G]) similar to PV_pv_ produced with the WT replicon (66, 67). These results suggested that replication of defective PV replicons could be efficiently rescued in *trans* and could allow production of a high titer PV_pv_.

## DISCUSSION

The importance of viral proteins provided in *cis* for replication of PV was initially suggested from analysis of defective interfering (DI) particles (28), which have in-frame deletions in the capsid-coding region (P1 region) of the genome and retain an intact non-structural protein coding region (P2P3 region) (29). These results suggested that the functions of viral proteins encoded in the P2P3 region could not be complemented by exogenous viral proteins, thus in *trans*. The *cis* and *trans* roles of the viral proteins in replication were intensively studied in the 1990s by *trans* complementation (or *trans* rescue) of replication of defective PV mutants (30–33, 37, 68–73). Main conclusions drawn from these studies include (i) *trans* rescue of defective PV mutants is inefficient, and (ii) a large intact precursor of the non-structural proteins is required for replication. Besides viral proteins, conserved RNA structures, encoded in the P2P3 region, were identified, including the CRE, RNase L ciRNA, α, and β (26, 74–78). The CRE is required for replication in *cis* as the template for uridylylation of 3B (26, 79), confirming the *cis* role of the P2P3 region. These properties/roles of non-structural proteins and RNA structures in viral replication are generally conserved in picornavirus (80–84).

To elucidate the role of processing in viral replication, I established cell lines that could conditionally express a large precursor of PV non-structural proteins (2BC3ABCD) and performed *trans* rescue of defective PV replicons in cells instead of using helper virus/replicon (Fig. 1). One major advantage of using these cell lines is the high expression level of the precursor protein in the presence of inhibitors against viral proteins and controllable expression for a short period to avoid cytotoxicity caused by viral proteases 3C^pro^/3CD^pro^ (85, 86). Processing of the 2BC3ABCD precursor gave both final and intermediate products, similar to those observed in PV-infected cells (27) (Fig. 1 and 8). I found that 2BC3ABCD could produce 2BC, 2B, 2C^ATPase/hel^, and 3AB (thus, also 3CD^pro^) by 3CD^pro^ provided in *trans,* but the processing of 3AB could occur only in *cis* (Fig. 8). In the *trans* rescue using the cell lines, replication was not detected for most of the mutants examined, except for a mutant with a disrupted cleavage site between 3C^pro^ and 3D^pol^ (3C/D[A/G] mutant), which showed a comparable level of replication to that of the WT replicon (Fig. 2 and 3). This allowed further analysis of the *cis* role of viral proteins in this mutant and revealed that only the activity of 3D^pol^, but not those of 3B, 2C^ATPase/hel^, or 3C^pro^, could be *trans* rescued (Fig. 4). PV 3CD^pro^ lacks RNA polymerase activity (87, 88); thus, the 3C/D(A/G) mutant, which could express only 3CD^pro^ but not 3C^pro^ nor 3D^pol^, was predicted to be deficient in polymerase activity irrespective of the introduction of inactivating mutations for 3D^pol^ activity (Fig. 4). Previous reports suggested that 3D^pol^ activity could be rescued in *trans* but with low efficiencies, similar to the rescue of 2C^ATPase/hel^ activity (30, 68, 69). The high expression level of the viral proteins in the cell lines might have improved the efficiency of the rescue and provided all-or-none replication. *trans* complementation of a PV mutant (EG mutant) with a partially defective cleavage site between 3B and 3C^pro^ has been reported (89), but I could not detect significant replication or *trans*-rescued replication of the corresponding mutant (3B/C[A/G] mutant, Fig. 2 and 3). This might suggest that partial or inefficient *cis* cleavage between 3B and 3C^pro^ is sufficient for the *trans-*rescued replication. Studies on foot-and-mouth disease virus (FMDV), which belongs to the genus *Aphthovirus* in the family *Picornaviridae* (90, 91), suggested that the defects in 3B uridylylation and 3D^pol^ activity could be rescued in *trans* (34, 35). FMDV is unique in coding multiple copies of 3B (three tandem copies of 3B). In addition, the recently discovered mosavirus has two copies of 3B (92), which could not be stably maintained in the PV genome (93). FMDV does not depend on host PI4KB/OSBP family I pathway for replication (94) in contrast to EVs, thus the mechanism and/or the role of uridylylation of 3B might be different from those in PV replication, in terms of *trans* rescue. Collectively, these results suggest a conserved *trans* role of 3D^pol^ activity in picornavirus replication.

**Fig 12:**
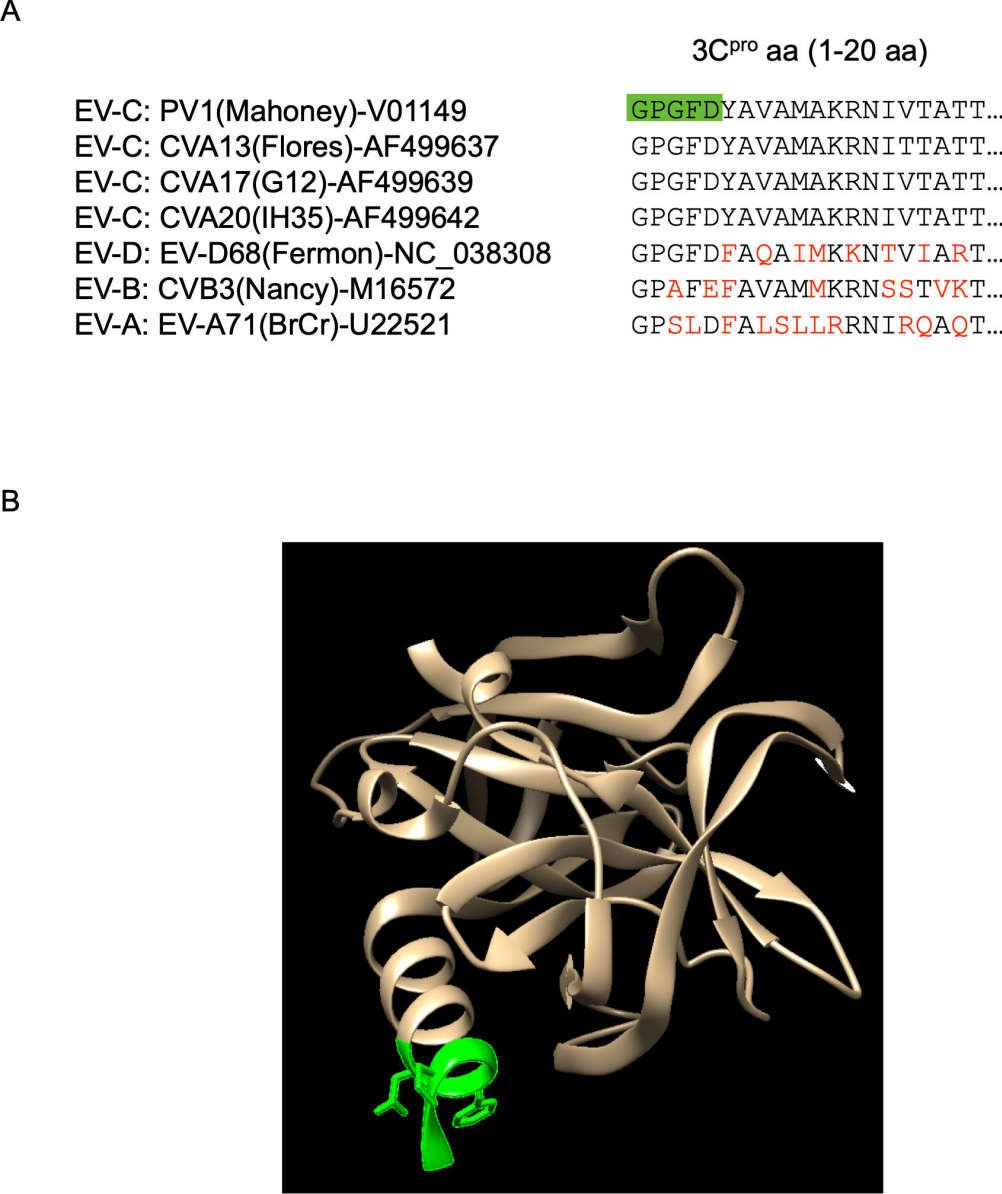
Comparison of primary and tertiary structures of 3C^pro^. (**A**) Sequence of aa 1–20 of 3C^pro^ of enterovirus. Difference of aa from that of PV1(Mahoney) is highlighted in red. The aa 1–5 of PV1(Mahoney) is highlighted in green. (**B**) Tertiary structure of PV 3C^pro^ (PDB: 4dcd). The aa 1–5 is highlighted in green.

**Fig 13:**
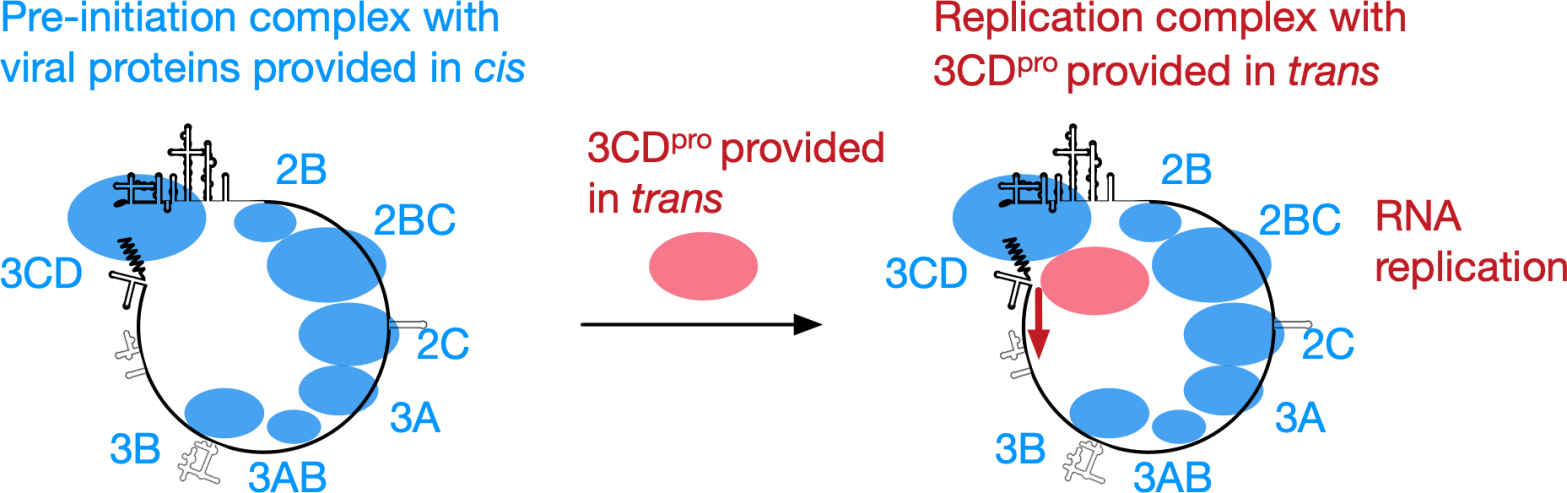
A model of PV replication by 3CD^pro^ provided in *trans*. The 3CD^pro^ provided in *trans* extends 3D^pol^ activity to the pre-initiation complex via virus species-specific protein–protein interactions.

To provide *trans* 3D^pol^ activity, expression of 3CD^pro^, but not of 3D^pol^, was essential and sufficient (Fig. 5 and 6); a large precursor was not necessarily essential in *trans* rescue. Unexpectedly, substitution of aa residues of 3C^pro^, which are involved in binding to viral RNA elements or phospholipids (4, 24, 26, 62), did not abrogate the *trans-*active function. This is in contrast to requirements for 3CD^pro^ in an *in vitro trans* complementation system; the interaction with RNA was essential for RNA synthesis and virus maturation (95, 96). This may suggest a critical difference between the pre-initiation complexes formed *in vitro* and *in vivo*, which could be rescued in *trans* by the 3CD^pol^ activity, possibly via a different pathway. I found that the N-terminal region of 3C^pro^ is essential for *in vivo trans* rescue (Fig. 8). Primary structures of the N-terminal region of 3C^pro^, which protrudes outside in the tertiary structure, are conserved within the viral species (53) (Fig. 12). PV non-structural proteins did not rescue a defective EV-A71 replicon in *trans*, suggesting that the interaction is dependent on the viral species (Fig. 7). I propose a model in which the 3CD^pro^ provided in *trans* provides 3D^pol^ activity to the viral pre-initiation complex, formed by the defective replicons, via virus species-specific protein–protein interactions (80) (Fig. 13).

Based on the observations of DI particles, the capsid-coding region is dispensable for the replication of PV; PV replicons coding exogenous reporter genes in place of the capsid-coding region have been developed (24, 97). Subsequently, generation of PV_pv_ that encapsidates engineered replicon with PV capsid proteins provided in *trans* has been established by using helper PV (98, 99), recombinant vaccinia virus systems (100, 101), or virus-free capsid-protein expression plasmid vector (65). PV_pv_ could serve as a safe alternative to live PV in biological tests, because no infectious virus is produced in the infected cells (65, 102). A limitation of these methods is that only replication-competent replicon RNA could be encapsidated (98, 103). In the present study, I partially solve this limitation to produce PV_pv_ with a defective PV replicon after *trans*-rescued replication (Fig. 10 and 11). This new generation of PV_pv_ may be useful for biological tests with enhanced safety.

The limitations of this study include the detection limits of *trans*-rescued replication; defective replicons give signals of reporters (firefly luciferase or mCherry) derived from initial protein synthesis before replication (about 1/10^2^ or 1/10^4^ of the signals at plateau, in the RNA-transfected cells or in PV_pv_-infected cells, respectively). Therefore, inefficient initial replication resulting only in quasi infection (70) might be missed in this study. In processing of a polyprotein (2BC3ABCD), only the processing of 3AB could occur in *cis*. However, a requirement for 2A^pro^, which can be deleted from the genome but requires a host factor SETD3 (104, 105), in *cis* processing remains to be elucidated.

Collectively, this work reveals potential roles of exogenous viral proteins in PV replication and offers insights into protein/protein interactions during picornavirus infection. This will aid in elucidating the mechanism of multiple PV infection, including intra-species recombination that can reduce the effectiveness of novel PV vaccines toward eradication (106, 107). Our findings might be useful for the development of effective antivirals targeting the polyprotein processing.

## MATERIALS AND METHODS

### Cells

RD cells (human rhabdomyosarcoma cells) and HEK293 cells (human embryonic kidney cells) were cultured as monolayers in Dulbecco’s modified Eagle medium (DMEM) supplemented with 10% fetal calf serum (FCS). RD cells were used for virus titration. HEK293 cells were used for expression of PV non-structural proteins and for production of PV_pv_.

### Viruses

PV_pv_ was produced with a firefly luciferase-coding or an mCherry-coding type 1 PV (PV1) Mahoney strain (GenBank: V01149) replicon and capsid proteins of PV1(Mahoney)(PV1[Fluc]_pv_ or PV1[mCherry]_pv_, respectively) (65).

### Chemicals

DOX was purchased from FUJIFILM Wako Pure Chemical Corporation (049-31121). GuHCl (a 2C inhibitor) was purchased from Sigma (G-9284). GC376 (a 3C inhibitor) was purchased from Selleck Chemicals (S0475). Rupintrivir (a 3C inhibitor) was purchased from Santa Cruz Biotechnology (sc-208317). Stock solutions of DOX (2 g/L) and GuHCl (2 M) were prepared in Milli-Q water. Stock solutions of GC376 (100 mM) and rupintrivir (10 mM) were prepared in dimethyl sulfoxide.

### General methods for molecular cloning

*Escherichia coli* strain XL10gold (Stratagene) was used for the preparation of plasmids. Ligation of DNA fragments was performed using an In-Fusion HD Cloning Kit (Clontech). PCR was performed using KOD Plus DNA polymerase (Toyobo). DNA sequencing was performed using a BigDye Terminator v3.1 cycle sequencing ready reaction kit (Applied Biosystems) and then analyzed with a 3500xL genetic analyzer (Applied Biosystems).

### Plasmids

#### Lentivirus expression vectors for PV non-structural proteins

##### pTet-AG-PV-2B2CP3(WT)

A cDNA fragment of a polyprotein of PV non-structural proteins (2BC3ABCD) was obtained by PCR with pPV-Fluc mc (a plasmid encoding cDNA of a PV replicon) (108) as the template and following primer set 1. This DNA fragment was inserted into a DNA fragment of a lentivirus vector plasmid with a TRE3G promoter, which was obtained by PCR with pLJM1-TRE3G-His-AG-FLAG-PreScission-OSBP(406–807) (52) as the template and using primer set 2.

##### Primer set 1

5′ GAAGTTCTGTTCCAGGGCCTCACCAATTACATAGAGTCAC 3′

5′ TCTGAGTCCGGATCAAAATGAGTCAAGCCAACGGCGGTAC 3′

##### Primer set 2

5′ CTGGAACAGAACTTCCAGCTTGTCGTCATC 3′

5′ TGATCCGGACTCAGATCTCGAGCTCAAGC 3′

##### pTet-AG-PV-2B2CP3(3C-C147A)

Mutations for the 3C-C147A aa substitution (disruption of the catalytic triad of PV 3C at aa C147) (59) was introduced in pTet-AG-PV-2B2CP3(WT) by PCR with primer set 3.

##### Primer set 3

5′ ACCAGAGCAGGACAGGCUGGTGGAGTCATCACATGTACTG 3′

5′ CTGTCCTGCTCTGGTTGGAAAGTTGTAC 3′

##### pTet-AG-PV-3CD(WT) and pTet-AG-PV-3CD(∆4-5 aa)

A deletion of the 2BC3AB-coding region was introduced in pTet-AG-PV-2B2CP3(WT) by PCR with primer set 4. An unexpected deletion in the primer region, causing a deletion of aa 4 and 5 in 3C region, was found in a clone, which was designated as pTet-AG-PV-3CD(∆4-5 aa).

##### Primer set 4

5′ GAAGTTCTGTTCCAGGGACCAGGGTTCGATTACGCAGTGG 3′

5′ CTGGAACAGAACTTCCAGCTTGTCGTCATC 3′

##### pTet-AG-PV-3CD(3C-R13N, 3C-K82N, 3C-R84S)

Mutations for the aa substitutions that could affect interaction with negatively charged molecules (viral RNA and phospholipids) (3C-R13N, 3C-K82N, and 3C-R84S aa substitutions) (24, 62–64) were introduced in pTet-AG-PV-3CD(WT) by PCR with primer sets 5 ,6, and 7, respectively.

##### Primer set 5

5′ AACATTGTTACAGCAACTACTAGCAAG 3′

5′ TGCTGTAACAATGTTGTTTTTAGCCATAGCCACTGCGTAATCG 3′

##### Primer set 6

5′ TTCAGAGACATTAGACCACATATACC 3′

5′ TCTAATGTCTCTGAAGTTTTCATTTCTCTTTAGAGTGATTATAG 3′

##### Primer set 7

5′ GACATTAGACCACATATACCTACTC 3′

5′ ATGTGGTCTAATGTCGCTGAACTTTTCATTTCTCTTTAGAGTG 3′

##### pTet-AG-PV-3CD(WT-uncleavable AG, ∆4-5 aa-uncleavable AG)

A mutation that disrupts the cleavage site for the 3C protease between the AG and the 3CD was introduced in pTet-AG-PV-3CD(WT) or pTet-AG-PV-3CD(∆4-5 aa) by PCR with primer set 8.

##### Primer set 8

5′ GGACCAGGGTACGCAGTGGCTATGGC 3′

5′ TGCGTACCCTGGTCCGGCGAACAGAACTTCCAGCTTGTCGTC 3′

### PV replicon mutants

#### pPV-Fluc mc (2A/B[A/G], 2B/C[A/G], 2C/3A[A/G], 3A/B[A/G], 3B/C[A/G], and 3C/D[A/G]) and pPV-mCherry mc (3C/D[A/G])

Mutants with disrupted cleavage sites for the 3C protease: the mutations between each viral gene (2A/B[A/G], 2B/C[A/G], 2C/3A[A/G], 3A/B[A/G], 3B/C[A/G], and 3C/D[A/G] mutations) were introduced in pPV-Fluc mc with a hammerhead ribozyme at the 5′end of the replicon (109, 110) by PCR with primer sets 9, 10, 11, 12, 13, and 14, respectively. The 3C/D(A/G) mutation was also introduced in pPV-mCherry mc with a hammerhead ribozyme at the 5′end of the replicon (110) by PCR with primer set 14.

#### Primer set 9

5′ GGCCTCACCAATTACATAGAGTCACTTGGG 3′

5′ GTAATTGGTGAGGCCGGCTTCCATGGCTTC 3′

#### Primer set 10

5′ TATGTCATCAAGGCCGGTGACAGTTGGTTGAAGAAGTTTACTG 3′

5′ GGCCTTGATGACATAAGGTATCTCCAGAACATCGC 3′

#### Primer set 11

5′ GGACCACTCCAGTATAAAGACTTGAAAATTG 3′

5′ ATACTGGAGTGGTCCGGCAAACAAAGCCTCC 3′

#### Primer set 12

5′ CTGTTTGCTGGACACGCCGGAGCATACACTGGTTTACCAAAC 3′

5′ GGCGTGTCCAGCAAACAGTTTATACATGAC 3′

#### Primer set 13

5′ GGACCAGGGTTCGATTACGCAGTGGCTATG 3′

5′ ATCGAACCCTGGTCCGGCTACCTTTGCTGTC 3′

#### Primer set 14

5′ TTCACTCAGAGTGCCGGTGAAATCCAGTGGATGAGACCTTCG 3′

5′ GGCACTCTGAGTGAAGTATGATCGCTTCAGGG 3′

#### pPV-Fluc mc (∆2A, ∆2B, ∆2C, ∆3A, ∆3B, ∆3C, and ∆3D)

Mutants with deletion of each viral gene: deletion of each viral gene (∆2A, ∆2B, ∆2C, ∆3A, ∆3B, ∆3C, and ∆3D) was introduced in pPV-Fluc mc by PCR with primer sets 15, 16, 17, 18, 19, 20, and 21, respectively.

#### Primer set 15

5′ GAAGCCATGGAACAAGGCCTCACCAATTAC 3′

5′ TTGTTCCATGGCTTCTGTGGTGAGCTCCAATTTG 3′

#### Primer set 16

5′ GGTGACAGTTGGTTGAAGAAGTTTACTG 3′

5′ CAACCAACTGTCACCTTGTTCCATGGCTTCTTCTTCGTAGGCATAC 3′

#### Primer set 17

5′ GGACCACTCCAGTATAAAGACTTGAAAATTG 3′

5′ ATACTGGAGTGGTCCTTGCTTGATGACATAAGGTATCTCCAGAAC 3′

#### Primer set 18

5′ GGAGCATACACTGGTTTACCAAACAAAAAACC 3′

5′ ACCAGTGTATGCTCCTTGAAACAAAGCCTCCATACAATTGCCAATG 3′

#### Primer set 19

5′ GGACCAGGGTTCGATTACGCAGTGGCTATG 3′

5′ ATCGAACCCTGGTCCCTGGTGTCCAGCAAACAGTTTATACATGAC 3′

#### Primer set 20

5′ GGTGAAATCCAGTGGATGAGACCTTCGAAG 3′

5′ CCACTGGATTTCACCTTGTACCTTTGCTGTCCGAATGGTGGGCAC 3′

#### Primer set 21

5′ TAGTAACCCTACCTCAGTCGAATTGGATTG 3′

5′ GAGGTAGGGTTACTATTGACTCTGAGTGAAGTATGATCGCTTCAG 3′

### pPV-Fluc mc (3C/D[A/G-2C-K153A], 3C/D[A/G-3B-Y3F], and 3C/D[A/G-3D-D328N/D329N])

Mutants encoding functionally inactive viral proteins (2C, 3B, and 3D) with a disrupted cleavage site for 3C protease between 3C and 3D: functionally inactivating mutations in PV 2C (a 2C-K153A aa substitution) (6), PV 3B (a 3B-Y3F aa substitution) (58), and PV 3D (3D-D328N/D329N aa substitution) (60) were introduced in pPV-Fluc mc (3C/D[A/G]) by PCR with primer sets 22, 23, and 24, respectively.

#### Primer set 22

5′ CCCGGAACAGGTGCCTCTGTAGCAACCAACCTGATTGCTAG 3′

5′ GGCACCTGTTCCGGGGCTGCCATGTACTAGC 3′

#### Primer set 23

5′ ACACCAGGGAGCATTCACTGGTTTACCAAACAAAAAACCCAACG 3′

5′ ATGCTCCCTGGTGTCCAGCAAACAGTTTATAC 3′

#### Primer set 24

5′ AATAATGTAATTGCTTCCTACCCCCATGAAG 3′

5′ AGCAATTACATTATTACCATAGGCAATCATTTTTAGG 3′

### EV-A71 replicon mutants

#### pEV-A71-Fluc mc (WT and 3C/D[A/G] mutant)

A hammerhead ribozyme (109) was introduced in pEV71(Fluc-mc) (111) at the 5′end of an EV-A71 BrCr-TR strain (GenBank: AB204852) replicon coding firefly luciferase reporter by PCR with primer set 25.

##### Primer set 25

5′ CGGTATCCCGGGTTCTTAAAACAGCCTGTGGGTTGCACCC 3′

5′ GAACCCGGGATACCGGGTTTTCGGCCTTTCGGCCTCATCAGTTAAAACACCCTATAGTGAGTCGTATTAATACGTAATTTCG 3′

The mutations between 3C and 3D (3C/D[A/G] mutations) were introduced in the above plasmid by PCR with primer set 26.

##### Primer set 26

5′ GGCCTCGCTGGCAAAATAACTCCTCTTTAG 3′

5′ TTTGCCAGCGAGGCCGGAGAGATCCAGTGGATGAAGCCTAACAG 3′

### RNA transfection

RNA transcripts of PV replicons were obtained using a RiboMAX Express Large Scale RNA Production System (Promega, P1320) with *Dra*I-linearized plasmids of PV replicons. RNA transcripts (0.025 µL) were transfected into the cells (4 × 10^4^ cells per well in 100-µL medium) in a 96-well plate (Corning Incorporated, 3595) using *Trans*IT-mRNA Transfection Kit (Mirus, MIR 2250).

### Preparation of PV_pv_ with a defective PV replicon

Tet-AG-PV-3CD(WT) cells (1.6 × 10^6^ cells per well in 4 mL medium) in a six-well plate (Corning Incorporated, 3516) were transfected with 4µg of a PV1(Mahoney) capsid-expression vector (pKS435-EGFP-PV1[Mahoney]) (108) per well using *Trans*IT-PRO transfection kit (Mirus, MIR 5700) in the presence of DOX (1 mg/L) and GC376 (100 µM). The cells were incubated at 37°C for 24h and then washed with the medium without DOX and GC376. RNA transcripts (1 µL) of defective PV replicons (3C/D[A/G]) with mCherry or firefly luciferase reporters were transfected into monolayers of Tet-AG-PV-3CD(WT) cells transiently expressing the capsid proteins and the 3CD protein in the absence of DOX and GC376. The cells were harvested at 24h post-transfection of the RNA transcripts and then stored at −20°C.

### Titration of defective PV_pv_

For the titration of PV1(mCherry)_pv_ (3C/D[A/G]), Tet-AG-PV-3CD(WT) cells (4 × 10^4^ cells per well in 100-µL medium) or Tet-AG-PV-3CD(∆4-5 aa) cells (a negative control of the infection) in a 96-well plate (Corning Incorporated, 3595) were incubated at 37°C for 5 h in the presence of DOX (1 mg/L). The cells were inoculated with 10 µL of serially diluted PV1(mCherry)_pv_ (3C/D[A/G]) solution (dilution of 1/1 to 1/10^5^) and then incubated at 37°C for 17 h. IUs of PV1(mCherry)_pv_ (3C/D[A/G]) were determined by counting the number of the mCherry-fluorescence positive cells (44). Images were collected at 4× magnification using a BZ-9000 fluorescence microscopy (Keyence) and then analyzed by using CellProfiler software (112). For the titration of PV1(Fluc)_pv_(3C/D[A/G]), Tet-AG-PV-3CD(WT) cells (8 × 10^3^ cells per well in 20-µL medium) or Tet-AG-PV-3CD(∆4-5 aa) cells (a negative control of the infection) in a 384-well plate (Greiner Bio-One, 781080) were incubated at 37°C for 5 h in the presence of DOX (1 mg/L). The cells were inoculated with 5 µL of serially diluted PV1(Fluc)_pv_(3C/D[A/G]) solution (dilution of 1/1 to 1/10^5^) and then incubated at 37°C for 17 h. The supernatant (15 µL) was removed from each well, and then 10 µL of Steady-Glo Reagent (Promega, E2520) was added to each well. Luciferase signals were measured using a 2030 ARVO X luminometer (Perkin-Elmer).

### Neutralization of defective PV_pv_

PV1(mCherry)_pv_ (WT or 3C/D[A/G] mutant) (2.0 × 10^3^ IU in 10 µL medium) was mixed with 10 µL of standard anti-PV1, PV2, or PV3 antisera (26 U for each type of PV) at 4°C for 7 h. Tet-AG-PV-3CD(WT) cells (4 ⋅10^4^ cells per well in 100 µL medium) in a 96-well plate (Corning Incorporated, 3595) were incubated at 37°C for 5 h in the presence of DOX (1 mg/L). PV1(mCherry)_pv_ (WT or 3C/D[A/G] mutant) (2.0 × 10^3^ IU in 10 µL medium) was mixed with 10 µL of standard anti-PV1, PV2, or PV3 antisera (26 U for each type) at 4°C for 7 h, and then added to the DOX-treated Tet-AG-PV-3CD(WT) cells. The cells were incubated at 37°C for 17 h, and then the number of the mCherry-fluorescence positive cells was counted as described in the titration of PV1(mCherry)_pv_.

### Western blot

The cells (8 × 10^5^ cells) were collected in 20 µL of cell lysis buffer (21 mM HEPES buffer [pH 7.4], 0.7 mM disodium hydrogenphosphate, 137 mM NaCl, 4.8 mM KCl, 0.5% Nonidet P-40, and 5 mM EDTA, supplemented with complete-mini protease inhibitor cocktail tablet [Roche, 04 693 159 001]) and were subjected to e-PAGEL 5%–20% gradient polyacrylamide gel electrophoresis (Atto Corporation) in a Laemmli buffer system. Proteins in the gel were transferred to a polyvinylidene difluoride filter (Millipore, Immobilon) and were blocked in iBind solution (Thermo Fischer Scientific). Filters were incubated with anti-PV 2C, 3A, and 3D antibodies (37) (rabbit antisera, 1:500, 1:200, and 1:250 dilution, respectively), then with secondary antibodies (Thermo Fisher Scientific, 32460, goat anti-rabbit IgG antibodies conjugated with horseradish peroxidase, 1:200 dilution) in iBind Western System (Thermo Fischer Scientific). Signals were detected by SuperSignal West Femto Maximum Sensitivity Substrate (Thermo Fisher Scientific, 34095) and then analyzed with ImageQuant 800 (Cytiva).

### Statistical analysis

Results of experiments are shown as means with standard deviations. Presented data are representative of at least two independent experiments with two or three biological replicates. Values of *P* < 0.05 by one-tailed *t*-test were considered to indicate a significant difference and were indicated by asterisks (*P < 0.05, **P < 0.01, ***P < 0.001).
